# An Integrated Genomic and Transcriptomic Analysis Reveals Candidates of Susceptibility Genes for Crohn’s Disease in Japanese Populations

**DOI:** 10.1038/s41598-020-66951-5

**Published:** 2020-06-24

**Authors:** Yoichi Kakuta, Ryo Ichikawa, Yuta Fuyuno, Atsushi Hirano, Junji Umeno, Takehiro Torisu, Kazuhiro Watanabe, Akihiro Asakura, Takeru Nakano, Yasuhiro Izumiyama, Daisuke Okamoto, Takeo Naito, Rintaro Moroi, Masatake Kuroha, Yoshitake Kanazawa, Tomoya Kimura, Hisashi Shiga, Takeshi Naito, Motohiro Esaki, Yosuke Kawai, Katsushi Tokunaga, Minoru Nakamura, Takayuki Matsumoto, Masao Nagasaki, Yoshitaka Kinouchi, Michiaki Unno, Atsushi Masamune

**Affiliations:** 1 Division of Gastroenterology, Tohoku University Graduate School of Medicine, Sendai, Japan; 2 Department of Medicine and Clinical Science, Graduate School of Medical Sciences, Kyushu University, Fukuoka, Japan; 3 Department of Surgery, Tohoku University Graduate School of Medicine, Sendai, Japan; 4 Human Biosciences Unit for the Top Global Course Center for the Promotion of Interdisciplinary Education and Research (CPIER), Kyoto University, Kyoto, Japan; 5 Department of Endoscopic Diagnostics and Therapeutics, Saga University Hospital, Saga, Japan; 6 Genome Medical Science Project, National Center for Global Health and Medicine, Tokyo, Japan; 7 Clinical Research Center, National Hospital Organisation (NHO) Nagasaki Medical Center, Omura, Japan; 8 Division of Gastroenterology, Department of Internal Medicine, Faculty of Medicine, Iwate Medical University, Morioka, Japan; 9 Department of Integrative Genomics, Tohoku Medical Megabank Organization, Tohoku University, Sendai, Japan; 10 Student Health Care Center, Institute for Excellence in Higher Education, Tohoku University, Sendai, Japan

**Keywords:** Genetic association study, Crohn's disease, Gene expression

## Abstract

Expression quantitative trait locus (eQTL) analyses have enabled us to predict the function of disease susceptibility SNPs. However, eQTL for the effector memory T cells (TEM) located in the lamina propria mononuclear cells (LPMCs), which play an important role in Crohn’s disease (CD), are not yet available. Thus, we conducted RNA sequencing and eQTL analyses of TEM cells located in the LPMCs from IBD patients (n = 20). Genome-wide association study (GWAS) was performed using genotyping data of 713 Japanese CD patients and 2,063 controls. We compared the results of GWAS and eQTL of TEM, and also performed a transcriptome-wide association study using eQTL from Genotype Tissue Expression project. By eQTL analyses of TEM, correlations of possible candidates were confirmed in 22,632 pairs and 2,463 genes. Among these candidates, 19 SNPs which showed significant correlation with *tenascin-XA* (*TNXA*) expression were significantly associated with CD in GWAS. By TWAS, *TNFSF15* (FDR = 1.35e-13) in whole blood, *ERV3-1* (FDR = 2.18e-2) in lymphocytes, and *ZNF713* (FDR = 3.04e-2) in the sigmoid colon was significantly associated with CD. By conducting integration analyses using GWAS and eQTL data, we confirmed multiple gene transcripts are involved in the development of CD.

## Introduction

Inflammatory bowel disease (IBD) is a term for two conditions: Crohn’s disease (CD) and ulcerative colitis. IBD is a multifactorial disease where development of the disease involves both hereditary factors and environmental factors. A genome-wide association study (GWAS) was conducted by various institutions to identify hereditary factors, which revealed that more than 200 regions in the human genome confer susceptibility to IBD^1–3^. However, many of the polymorphisms that show correlation with the disease are located in non-transcribed regions. Regions that show disease susceptibility due to functional mutation caused by amino acid substitution were limited to regions such as the *nucleotide binding oligomerization domain-containing 2* (*NOD2*), *interleukin 23 receptor* (*IL23R*), and *autophagy related 16-like 1* (*ATG16L1*). The GWAS of Japanese CD patients, reported by us in 2019, indicated that the only polymorphism with amino acid substitution among 11 identified disease susceptibility regions was the p.G149R polymorphism located in *IL23R*^4^. In cases of polymorphisms with amino acid substitution, it is highly likely that genes with such polymorphisms are involved in the development of IBD. However, the mechanism of IBD development caused by other polymorphisms is unknown, and susceptibility genes remain to be confirmed. It is predicted that the genomic mutations located in these disease susceptibility regions impact the expression of nearby genes and are involved in the development of IBD.

In recent years, many expression quantitative trait locus (eQTL) analyses have been performed with the aim of examining the relationship between comprehensive gene expression in various cell types and the genetic background. These findings have been used to create a database. Within this database, the Genotype Tissue Expression (GTEx) project examined gene polymorphism expression of every human tissue^5^. Using the eQTL database, it is possible to predict which tissues are affected by gene polymorphisms, which genes are involved, and what is the degree of expression of these genes. Furthermore, it is now possible to examine the relationship between polymorphism and changes in expression, to predict changes in gene expression levels caused by polymorphisms, and to perform a transcriptome-wide association study (TWAS) based on the data^6^.

By utilizing GWAS and eQTL analysis, polymorphisms that correlate to the development of IBD can be identified and the expression of genes impacted by these polymorphisms can be predicted. Moreover, TWAS enabled us to predict disease susceptibility genes and changes in expression that cause IBD by analyzing each gene unit.

However, each eQTL database is an analysis performed under specific conditions in specific cells. Moreover, racial variations need to be considered. To determine the causes of the development of CD, it is important to consider gene expression and the relationship of gene expression to single-nucleotide polymorphisms (SNPs) in cells that play a role in immunity in the sites of inflammation of the disease (i.e., the intestinal tissues). Although data regarding samples such as the small intestine, large intestine, and whole blood are available from previously described GTEx, data for the immunocompetent cells located in the intestinal sites are not yet available. Thus, the genes involved in IBD and how the expression of such genes is impacted by susceptibility gene polymorphism in Japanese IBD patients remain unknown.

Based on the above, we performed eQTL analyses by collecting CD4+ effector memory T cells (TEM cells) from lamina propria mononuclear cells (LPMCs), the cell type considered to be involved in disease state of Japanese CD patients. Using our results and the eQTL data from previously constructed database for other tissues, disease susceptibility genes involved in the development of CD in the Japanese population were identified.

## Results

### In LPMC-derived TEM cells, eQTL of 2,463 genes at 22,632 regions were identified

**Figure 1:**
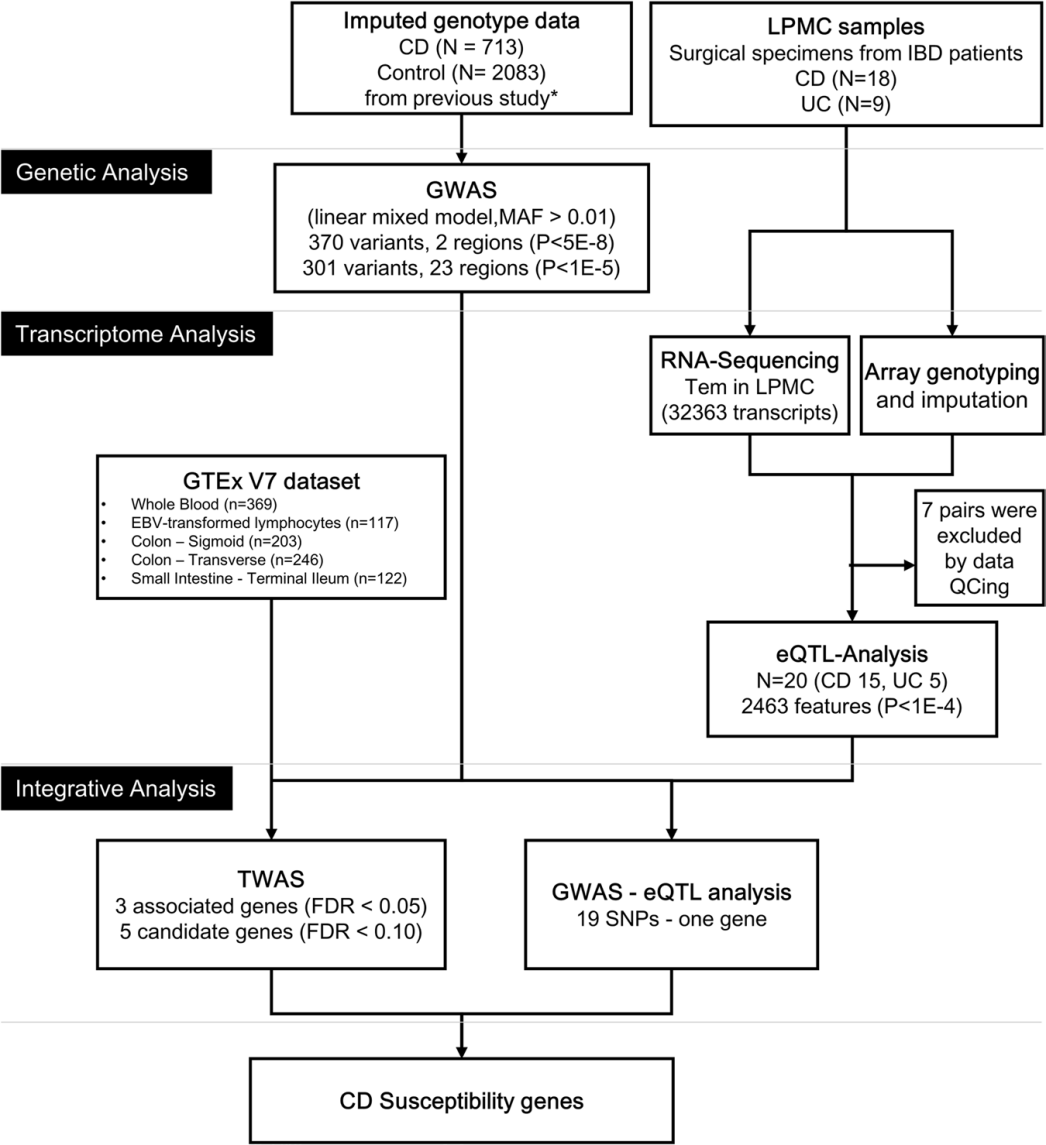
Analytical flow in this study. CD; Crohn’s disease, UC; ulcerative colitis, LPMC; lamina propria mononuclear cells, GWAS; genome-wide association study, MAF; Minor allele frequency, GTEx; Genotype Tissue Expression, EBV; Epstein–Barr virus, eQTL; expression quantitative trait locus, TWAS; transcriptome-wide association study, FDR; False Discovery Rate.

The analysis flow chart is shown in Fig. 1. RNA sequencing performed on TEM cells of 20 IBD patients (15 CD patients, 5 UC patients), which advanced to expression analysis, confirmed expression of 32,363 genes. According to eQTL analyses, 22,632 pairs in 2,463 genes were confirmed to be candidates (*p* < 1e-04) which showed correlation between gene polymorphism and expression. Among these pairs, 2,000 pairs in 220 genes showed significant (*p* < 1e-06) correlation (Supplementary Tables S1 and S2).

### Twenty-five sites were confirmed as candidates correlated with Japanese CD by GWAS

Manhattan plots were constructed based on the GWAS of CD patients performed using a linear mixed model (Supplementary Fig. S1). Significant correlation was found in 370 SNPs (*p* < 5e-08). These SNPs were found to be located in two regions, the human leukocyte antigen (HLA) region on chromosome 6 (rs184950714, *p* = 1.07e-17) and upstream of *tumor necrosis factor superfamily member 15* (*TNFSF15*) (rs55951892, *p* = 1.76e-23) on chromosome 9. Moreover, 301 SNPs that showed a candidate level of correlation (p < 1e-05) were found in an additional 23 regions (Table 1). Among the SNPs that showed more than a candidate level of correlation, only three polymorphisms, IL23R p.Gly149Arg (*p* = 4.22e-07), IL27 p.Leu119Pro (*p* = 3.28e-05), and SULT1A2 p.Asn235Thr (*p* = 4.38e-05), showed amino acid substitutions (Supplementary Table S3).

**Table 1:** Summary of the CD-GWAS results in Japanese patients.

Chr	Range (bp)*		Top Hit SNP							No. of SNPs**	Genes
	From	To	SNP ID	Position*	A1	A2	A2 Frequencies	P-Values	OR (95%CI)		
1	20437634	20437764	rs7515774	20437634	A	T	0.107	1.04E-06	1.10 (1.06–1.15)	2	*PLA2G2D*
1	67648596	67648596	rs76418789	67648596	G	A	0.060	4.22E-07	0.88 (0.84–0.93)	1	*IL23R* (G149R)
1	112222702	112222702	rs534888	112222702	C	T	0.467	7.16E-06	0.95 (0.92–0.97)	1	*RAP1A*
4	38324507	38373273	rs55843528	38361416	G	A	0.245	1.89E-07	1.08 (1.05–1.11)	20	—
4	189893313	189893313	rs12647478	189893313	T	C	0.391	7.58E-06	0.92 (0.88–0.95)	1	—
5	67691469	67691469	rs10068082	67691469	G	A	0.037	1.72E-06	1.17 (1.10–1.26)	1	*PIK3R1*
5	158826792	158853941	rs56167332	158827769	C	A	0.402	3.11E-07	1.07 (1.04–1.09)	21	*IL12B*
6	2957827	2966578	rs79536569	2957827	G	A	0.017	3.50E-06	1.23 (1.13–1.35)	2	*SERPINB6*
**6**	**32214010**	**32793981**	**rs184950714**	**32636728**	**G**	**A**	**0.179**	**1.07E-17**	**1.16 (1.12–1.19)**	**452**	**(HLA)**
7	12221801	12222116	rs200319458	12221801	T	C	0.024	8.41E-06	1.19 (1.10–1.29)	2	*TMEM106B*
7	76948351	76948351	rs4727354	76948351	G	T	0.051	3.31E-06	1.14 (1.08–1.20)	1	*GSAP*
8	70472493	70472493	rs117742432	70472493	G	A	0.036	6.58E-06	0.86 (0.81–0.92)	1	*SULF1*
8	129224694	129245849	rs12678162	129224694	T	C	0.431	3.08E-06	1.06 (1.03–1.08)	4	*PVT1*
9	73881874	73881874	rs151258497	73881874	—	C	0.331	4.57E-06	0.94 (0.92–0.97)	1	*TRPM3*
**9**	**117480416**	**117697947**	**rs55951892**	**117575913**	**A**	**C**	**0.490**	**1.76E-23**	**0.89 (0.86**–**0.91)**	**129**	***TNFSF15***
10	64431973	64550071	rs224136	64470675	C	T	0.275	1.18E-07	0.93 (0.90–0.95)	19	*ZNF365*
11	64908062	64926722	rs11227126	64908062	A	T	0.067	1.71E-06	1.12 (1.07–1.18)	3	*SYVN1*
14	105695957	105695957	rs117952084	105695957	T	C	0.021	9.73E-06	0.83 (0.76–0.90)	1	*BRF1*
15	27156539	27156539	rs781387485	27156539	ACACAA	—	0.095	3.36E-06	1.11 (1.06–1.16)	1	*GABRA5*
16	28513068	28531287	rs56354901	28523144	T	C	0.134	2.44E-06	1.09 (1.05–1.13)	3	*NPIPL1, IL27*
19	30021446	30021446	rs117223925	30021446	G	A	0.075	6.21E-06	0.90 (0.86–0.94)	1	*VSTM2B*
20	47804952	47804952	NA	47804952	—	CCCGGC	0.020	2.57E-06	1.21 (1.12–1.32)	1	*STAU1*
20	51548302	51548302	rs6126698	51548302	A	T	0.484	8.31E-06	0.94 (0.91–0.97)	1	*TSHZ2*
21	33326781	33326781	rs2833577	33326781	G	A	0.286	1.23E-06	1.07 (1.04–1.11)	1	*HUNK*
22	23054614	23054614	rs9623882	23054614	G	A	0.081	1.61E-06	1.14 (1.08–1.20)	1	*GGTLC2*

^*^Positions are based on the Genome Reference Consortium human build 37 (GRCh37), **Number of SNPs with p values <1 × 10^−5^ Chr: Chromosome, OR: Odds ratio, CI: Confidential interval.

### Correlation between Japanese CD and *TNXA* based on GWAS and eQTL results was assessed

**Figure 2:**
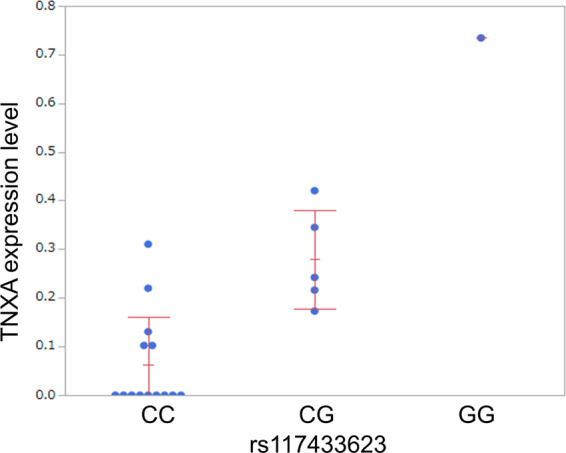
Relationship between rs117433623 and expression of *tenascin-XA* (*TNXA*) according to integration analysis of GWAS + eQTL. − Based on eQTL data confirmed in this study, correlation between the expression of 19 SNPs on chromosome 6 in candidate polymorphisms and expression of *TNXA* in intestinal TEM cells were identified. GWAS; genome-wide association study, eQTL; expression quantitative trait locus.

Among the candidate polymorphisms identified by the GWAS, 19 SNPs of chromosome 6 showed significant correlation with expression of the *tenascin-XA* (*TNXA*) in intestinal TEM cells (rs117433623, *P*_*GWAS*_ = 6.34e-09, *P*_*eQTL*_ = 3.49e-05) (Fig. 2, Supplementary Figure S2, Supplementary Table S4). Only one SNP showed a genotype of GG; therefore, further analyses were conducted using two groups—CC and G carrier—in which a correlation tendency was also observed (*p* = 1.60e-03, Wilcoxon rank-sum test).

### Six novel genes were identified by TWAS in addition to the previously reported *TNFSF15* and *RAP1A*

**Figure 3:**
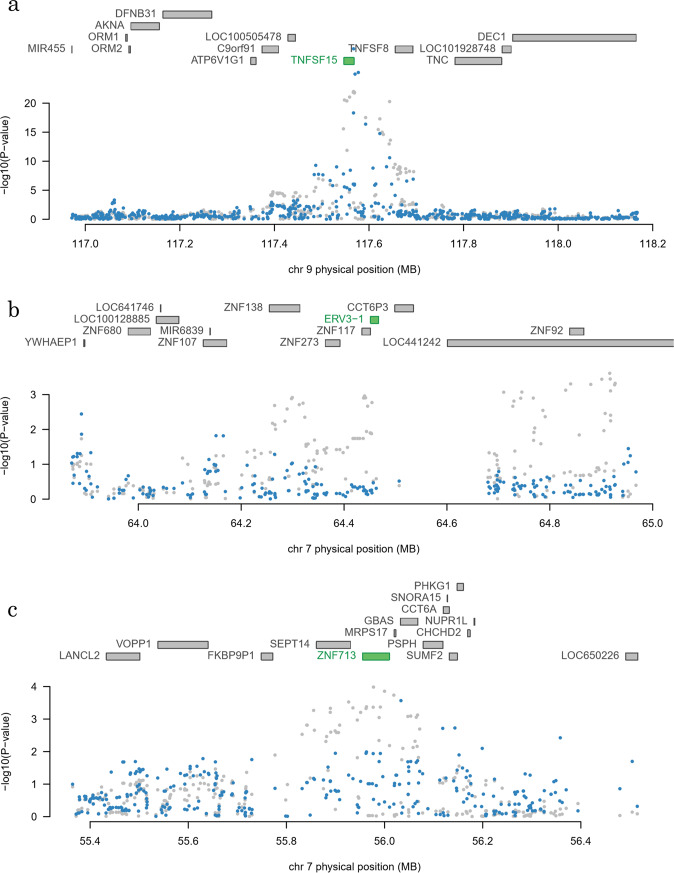
Correlation plots of SNPs in regions that showed significant correlation by TWAS. Figure shows plots of polymorphism periphery of eight genes (**a**. *TNFSF15*, **b**. *ERV3-1*, and **c**. *ZNF713*) that showed significant correlation by TWAS. Each dot indicates -log10(*p-values*) before (gray) and after (blue) adjustment by gene (green) that showed correlation. *ERV3-1* and *ZNF713* lose correlation after adjusting by genes showing correlation, although the correlation of *TNFSF15* remains.

Analyses of HLA regions by TWAS were performed separately from other regions. The relationship of gene expression of multiple genes such as HLA-DQ and HLA-DR with CD was confirmed in all analyzed cell types. Almost all of the correlations were found to be related to re9271170 in the GWAS (Supplementary Table S5). Excluding the HLA region, *TNFSF15* (TWAS. *p* = 2.28e-17, FDR = 1.35e-13) in whole blood, *endogenous retrovirus group 3 member 1* (*ERV3-1*) (TWAS. *p* = 4.79e-05, FDR = 2.20e-02) in EBV-immortalized lymphocytes, and *zinc finger protein 713* (*ZNF713*) (TWAS. *p* = 4.41e-05, FDR = 3.03e-02) in the sigmoidal colon showed significant correlation (Table 2). Additionally, *apolipoprotein B MRNA editing enzyme catalytic subunit 3 A* (*APOBEC3A*) in whole blood, *ras-related protein Rap-1A (RAP1A)* in EBV-immortalized lymphocytes, *nuclear pore complex interacting protein family member B9* (*NPIPB9*) and *immunoglobulin lambda variable 3-29* (*IGLV3-29*) in the transverse colon, and *WD repeat domain 31* (*WDR31*) in the sigmoidal colon showed possible associations (FDR < 0.10) as candidate genes (Table 2). Some of these genes showed possible associations in other tissues (Supplementary Table S6). Among these genes, correlation of SNPs within the regions of genes such as *ERV3-1, RAP1A, ZNF713* was lost when a correction was made using the predicted expression levels, however, multiple SNPs continued to show a strong correlation in *TNFSF15* after correction using the predicted expression levels (Fig. 3, Supplementary Figure S3).

**Table 2:** Summary of TWAS with the susceptibility genes for CD in Japanese patients (non-HLA genes).

Tissue	Gene	Chr	GWAS		eQTL			TWAS		
			Best SNP	Z-Score	SNP	R2	Z-Score	Z-Score	P-Value	FDR
**Significantly associated genes (FDR** < **0.05)**										
Whole blood	*TNFSF15*	9	rs4979462	9.81	rs7866342	6.47E-02	5.12	−8.48	2.28E-17	1.35E-13
Blood - EBV-transformed lymphocytes	*ERV3-1*	7	rs4718244	−3.67	rs4718244	8.28E-02	3.90	−4.07	4.79E-05	2.18E-02
Colon – Sigmoid	*ZNF713*	7	rs6971250	−3.88	rs6593287	1.06E-01	5.31	4.08	4.41E-05	3.04E-02
**Candidate genes (FDR** < **0.10)**										
Whole blood	*APOBEC3A*	22	rs5750616	−3.02	rs4821843	2.14E-02	−4.71	3.90	9.66E-05	6.79E-02
Colon - Transverse	*NPIPB9*	16	rs4788076	4.16	rs17640009	5.35E-02	−4.60	−3.92	8.74E-05	7.66E-02
Colon - Sigmoid	*WDR31*	9	rs10981725	−4.38	rs10817477	1.70E-01	−5.96	3.82	1.34E-04	8.07E-02
Blood - EBV-transformed lymphocytes	*RAP1A*	1	rs2786991	−4.30	rs530801	9.98E-04	3.48	3.67	2.42E-04	9.45E-02
Colon - Transverse	*IGLV3-29*	22	rs9623882	4.80	rs8140385	3.58E-02	4.31	3.83	1.29E-04	9.70E-02

Chr: Chromosome, GWAS: genome-wide association study, TWAS: transcriptome-wide association study, FDR: false discovery rate

## Discussion

The novel outcomes of this study were as follows: (1) even though on a small scale, eQTL data of intestinal LPMCs derived from the TEM cells of Japanese IBD patients were constructed for the first time, (2) polymorphisms that showed correlation by GWAS of Japanese CD patients indicated correlation with expression of *TNXA* in intestinal LPMC-derived TEM cells, (3) *TNFSF15* in whole blood and *RAP1A* in lymphocytes were confirmed to be disease susceptibility genes when using TWAS for the first time in Japanese CD patients, (4) six genes (including 4 candidates) were newly identified to be correlative.

The eQTL constructed in this study, albeit at a very small scale, was limited to intestinal LPMC-derived TEM cells of Japanese IBD patients and has not previously been reported. The reason why we analyzed eQTL in TEM cells was TEM is considered to be strongly associated with IBD pathogenesis. For example, colitis can be induced in immunodeficient mice by transferring naïve T cells^7^, strategies blocking T-cell function are useful for attenuating mucosal inflammation in mice with experimental colitis^8^, and IBD is frequently associated with other T-cell mediated diseases (i.e., psoriasis and multiple sclerosis)^9,10^. Based on the integration analysis of this eQTL data and the GWAS, new polymorphisms involved in the development of CD in the Japanese population that correlated to the expression of *TNXA* were identified. *TNXA* is considered a pseudogene which is not capable of producing functional protein. Therefore, it is unclear whether the gene is involved in the disease state, and if so, how it is involved. However, a report has suggested that TNXA is a serum protein characteristic of stricturing CD^11^. Thus, combined with this report, it is possible that *TNXA* actually codes for a protein with unknown function. In addition, it may be involved in the development of the specific disease phenotype of CD. However, there is currently insufficient data to conclude that an increased level of TNXA in the serum of CD patients is involved in the development of CD. Polymorphisms that showed correlation with CD may have two functions: one may involve the development of CD via other functions and the other may involve the expression of *TNXA* that does not code for functional protein. Hence, the expression of TNXA may function as a marker of polymorphism in the gene. Future studies should consider the function of TNXA using models (i.e., mice) in addition to the conformation of a *TNXA* expression level in intestinal sites in Japanese CD patients. Additionally, the association of *TNXA* gene with CD causality was shown indirectly by connecting the results of GWAS and eQTL. To confirm this association, additional analysis such as Mendelian randomization analysis with a larger eQTL data set of Tem from LPMC in the Japanese population should be performed.

In this study, TWAS was first conducted on Japanese CD patients with the use of previously reported eQTL data. A verified correlation around the periphery of *TNFSF15* may indicate disease susceptibility via *TNFSF15* expression in whole blood according to TWAS. In recent years, many statistical correlations of polymorphisms with unknown function have been identified because genome-wide studies has become available due to low-cost genome analysis technology. However, it is difficult to analyze the expression of genes of various tissue samples in terms of sample collection cost. On the other hand, eQTL databases of various cell types have been constructed and such databases have become freely available. TWAS is one approach that can be used to solve the limitations of GWAS by analyzing such databases integrally and is an analytical method that can be used to identify new disease susceptibility genes. Those regions sometimes contain multiple genes; however, correlation with each gene can be identified by TWAS due to the analysis of a gene unit. The correlation of the *TNFSF15* periphery identified by GWAS was found to exist in the region stretching from *TNFSF15* to *TNFSF8*; however, the whole region was indicated to be involved in *TNFSF15* expression and to correlate with CD, according to TWAS.

*TNFSF15* is a cytokine gene belonging to the TNF family (also called *TNF-like ligand 1 A* (*TL1A*)) and is known to show increased expression at intestinal CD sites^12^. TNFSF15 is mainly secreted from monocytic cells, such as macrophage and dendric cells, and is thought to promote Th1 and Th17 cell activities, leading to CD development^13^. Multiple studies have reported that *TNFSF15* polymorphisms involve gene expression^14,15^. TWAS results in this study agree with these reports. Therefore, the usefulness of TWAS is supported by analyses using independent databases such as TWAS.

The TWAS method used in this study confirmed multiple novel candidate genes in addition to *TNFSF15* and *RAP1A*^4,16,17^. APOBEC3A (cytidine deaminase) targets single-stranded DNA and functions as a restriction factor in retrovirus replication. It has been previously reported that this gene is involved in cell cycle arrest caused by DNA damage and oxidative stress^18^. Polymorphisms located relatively close to the gene are reported to correlate to IBD in the Western population; however, involvement of the genes in IBD has not been indicated. Therefore, this study showed such a correlation for the first time.

*ERV3-1* is a gene found in endogenous retroviruses; however, the relationship of *ERV3-1* with IBD has not been reported previously. The function of both *NPIPB9* and *IGLV3-29* is also unknown. One study has reported that changes in the expression level of *ZNF713* due to mutation in the gene are involved in autism spectrum disorder^19^; however, the function of ZNF713 and its relationship with IBD are unknown.

WDR31 is a member of the family of WD40 repeat proteins. WD40 repeat proteins belong to a large family observed among all eukaryotes and are involved in various functions, including signal transduction, regulation of transcription, regulation of cell cycle, autophagy, and apoptosis. It is plausible that changes in the expression of members belonging to this family of genes would relate to disease. In fact, *WDR30* is also known as *ATG16L1*, which is a disease susceptibility gene in Western CD patients and is involved in autophagy^20^. However, the function of *WDR31*, which showed correlation in this study, is currently unknown, and no relationship with IBD has been reported. Many of these novel candidate genes have unknown functions and unknown relationships with IBD; however, future functional analyses may provide this information. And these associations were only observed in colon, it will be interesting to see associations of these genes with each clinical sub-phenotype (i.e. disease locations) of CD. Further analyses using additional sample set will be needed.

This study showed that multiple correlations could be confirmed with the use of TWAS. Correlation shown by GWAS at the regions of some genes such as *ERV3-1* can be lost when a predicted expression level of ERV3-1 was taken into consideration. Therefore, it was indicated that correlation in the region is due to changes in the expression level of ERV3-1. However, correlation of some SNPs in genes such as *TNFSF15* does not diminish when a predicted expression level of the genes is taken into consideration; thus, it has been confirmed that some SNPs have correlation regardless of predicted gene expression levels. In fact, it has been demonstrated previously that there are two independent correlations in this region^21^, the result of which are consistent with those found this study. However, how the polymorphisms that showed independent correlations are involved in the disease is unknown. The referenced eQTL data are from the Western population, and there may be vastly distinctive Asian-specific eQTL data. Further research is necessary.

Limitations in this study regarding eQTL are as follows: (1) the sample size was small, (2) only IBD patients who required surgery were studied, and mild IBD patients who did not require surgery were not included in this study, (3) there were differences in inflammation sites and degree of inflammation in surgical specimens, and (4) there were difference in drugs administered before surgery (individual results may be affected by such drugs). Limitations of TWAS are that (6) referenced gene expression data are from a different ethnic group and (7) evaluation of genes induced under specific conditions was not possible. To increase the number of subjects and reduce the effect of medications or severity issue, analyses of biopsy samples at the initial endoscopy will be informative. However, we aimed to establish eQTL dataset of specific cell population in this study, we analyzed surgical specimens. The most serious limitation of our study was we could only see eQTLs of TEM cells in Japanese patients with IBD, because the number of LPMCs, which could be isolated from surgical specimens, was still too few to analyze several immunocompetent cells. The increasing number of samples and cell species of immunocompetent cells and/or adopting new technologies (i.e. single cell analysis) may show more certainly eQTL, although this is a subject for future analysis. However, this study included a functional approach utilizing data regarding function of polymorphisms in addition to existing GWAS, which simply examines whether SNPs are involved in the development of the disease. Factors related to the development of the disease at a gene level in a specific tissue could be predicted. Moreover, the results obtained in this study included genes (*TNFS15* and *RAP1A*) that have shown correlation by functional analyses as candidate genes and thus the usefulness of this approach was shown. Integration analyses using GWAS and eQTL data are considered useful not only for the analysis of disease susceptibility genes but also for analyzing disease-modifying genes that determine the disease state and pharmacogenomics, which involves analysis of drug efficacy and adverse effects. Future analyses are anticipated.

In conclusion, by conducting integration analyses using information regarding polymorphism and transcriptome-related analysis data, we confirmed multiple gene transcripts involved in the development of CD in the Japanese population. The study also indicated that expression of *TNFSF15* in blood cells was likely to be involved in the development of CD in the Japanese population.

## Materials and Methods

In this study, analyses were processed using the following two approaches to accomplish our objective. First, eQTL analyses were conducted on intestinal TEM cells of Japanese CD patients and disease susceptibility genes were predicted by projecting the function of disease susceptibility polymorphisms in these patients. Second, a TWAS was conducted using data from the existing eQTL database and the GWAS results to analyze the susceptibility genes of Japanese CD patients.

### Subjects

For TEM transcriptome analyses, cells were isolated from 18 patients who were in an active phase of CD and nine patients who were in an active phase of UC from a cohort of IBD patients hospitalized in Tohoku University Hospital between July 2015 and July 2018. The studied cohort underwent surgery that involved intestinal resection and consented to research including genetic analysis. The subjects for GWAS were 713 Japanese CD patients who regularly visited either Tohoku University Hospital (379 patients) or Kyushu University Hospital (334 patients) and could be analyzed by previous GWAS of Crohn’s disease^4^. A total of 2,063 healthy individuals who resided in Tohoku (1,621 individuals) or Kyushu (462 individuals) were also studied as controls^22^. Diagnosis was performed according to the diagnostic criteria proposed by the Japanese Ministry of Health, Labor and Welfare^23^, based on clinical symptoms and endoscopic, X-ray, and tissue findings. All subjects were Japanese.

This study was conducted after receiving written consent from subjects and approval from the ethics committee of the School of Medicine at Tohoku University (2017-1-253, 2019-1-161). All methods in this study were performed in accordance with ethical guidelines for medical and health research involving human subjects established by the Ministry of Health, Labour and Welfare in Japan. The demographic profiles of the subjects are shown in Table 3.

**Table 3:** Patient characteristics.

Sample	Disease	Age	Sex	Disease Location	Sampling site	Medication (active intervention)
IBD1	CD	36	M	ileum	ileum	5ASA
IBD2	CD	18	M	ileum	ileum	ADA
IBD3	CD	27	M	ileum	ileum	None
IBD4	CD	21	M	ileocolon	ileum	5ASA, UST, AZA
IBD5	CD	35	M	ileocolon	ileum	5ASA, IFX
IBD6	CD	40	M	ileocolon	ileum	5ASA
IBD7	CD	48	F	ileocolon	ileum	5ASA, ADA, AZA
IBD8	CD	26	M	ileocolon	ileum	5ASA
IBD9	CD	58	M	ileocolon	ileum	5ASA
IBD10	CD	40	M	ileocolon	ileum	5ASA, IFX
IBD11	CD	28	M	ileocolon	colon	5ASA, IFX, AZA
IBD12	CD	40	M	ileocolon	colon	5ASA, IFX
IBD13	CD	19	M	ileocolon	colon	IFX, 6MP
IBD14	CD	48	M	ileocolon	colon	5ASA, ADA, AZA
IBD15	CD	42	M	ileocolon	colon	5ASA
IBD16	UC	65	F	pancolitis	colon	5ASA, PSL, ADA
IBD17	UC	26	M	pancolitis	colon	PSL, IFX
IBD18	UC	75	F	pancolitis	colon	5ASA, PSL
IBD19	UC	49	F	pancolitis	colon	5ASA, PSL, AZA
IBD20	UC	65	M	pancolitis	colon	5ASA, PSL, Tac

CD: Crohn’s disease, UC: ulcerative colitis, 5ASA: 5 aminosalicylic acid, IFX: infliximab, ADA: adalimumab, UST: ustekinumab, AZA: azathiopurine, 6MP: 6 mercaptopurine, PSL: prednisolone, Tac: Tacrolimus

### Isolation of LPMCs

LPMCs were isolated from inflammation sites surgically resected from the small intestine or the large intestine according to the method described by Fiocchi *et al*.^24,25^. In brief, a resected specimen was cut lengthwise, and feces were removed by washing the intestine in Hank’s balanced salt solution (HBSS) (Wako, Osaka, Japan). The specimen was then cut into 2–3 cm × 10 cm sections. The sections were then washed in HBSS containing 0.15% dithiothreitol (Wako) for 30 minutes with shaking. The specimens were then washed in HBSS containing 1 mM ethylenediaminetetraacetic acid (Wako) for 90 minutes with shaking. This wash was repeated until the epithelial layer was completely removed. After removing the epithelial layer completely, the specimens were washed again in HBSS with shaking and the washed specimens were finely divided into 5-mm sections. The specimens were then digested in HBSS containing 1 mg/ml collagenase-3 (Worthington Biochemical Corporation, Lakewood, USA) and DNase I (Roche, Basel, Switzerland) at 37 °C for 8–10 hours. The digested specimens were then passed through a 100 μm cell strainer (BD Biosciences, Franklin Lake, USA) and the cell suspension was recovered. The suspension was centrifuged at 700 × *g* and the cell pellet was resuspended in HBSS. The suspension was overlaid on Ficoll–Hypaque (GE Healthcare, Little Chalfont, UK) and centrifuged for 20 minutes at 1,000 × *g*. LPMC cells located at the interface between HBSS and Ficoll–Hypaque were recovered.

### Isolation of TEM cells and extraction of DNA/RNA

CD4+ T cells were isolated by negative selection from isolated LPMCs using an Easy Sep Magnet (STEMCELL Technology, Vancouver, Canada) and an Easy Sep Human CD4+ T cell Enrichment kit (STEMCELL Technology). Furthermore, the isolated CD4 positive T cells were stained with anti-CD3-FITC, anti-CD4-PE, anti- CD45RO-APC, anti-CD197 (CCR7) -BV421, and 7ADD-Cell Viability Solution (BD Biosciences), followed by isolation of TEM cells using a FACS Aria II cell sorter (BD Biosciences). Sorting efficiency was consistently over 98%. These TEM cells may include a few regulatory T Cells. However, to keep the number of cells to perform RNA sequencing, we used these samples as TEM cells. DNA and total RNA were extracted from isolated TEM cells using an AllPrep DNA/RNA mini kit (QIAGEN, Hilden, Germany).

### Genotyping

Transcriptome analysis of subjects by Japonica array V1 (Thermo-Fisher Scientific Inc., Waltham, MA) was contracted to Toshiba Inc. (Tokyo, Japan)^26^. Affymetrix Power Tools software (Thermo-Fisher Scientific Inc.) was used for genotyping. For genotyping of SNPs that could not be typed by the array, IMPUTE2 (Version 2.3.2) (Center for Statistical Genetics, University of Michigan, USA) was used for performing imputation with the genome reference panel of people from the Tohoku region (2KJPN)^27,28^. For genotyping data for the GWAS, data which had undergone analyses by Japonica array VI, imputation by the 1KJPN panel, and quality control (QC) by previous studies were used^4^.

### Transcriptome and eQTL analyses

For the total RNA collected from the intestines of 27 IBD patients (18 CD patients, 9 UC patients), QC, library construction, and transcriptome analysis by RNA sequencing were contracted to Macrogen Inc, Japan. QC was performed using TapeStation HighSensitivity RNA ScreenTape (Agilent Technologies, Santa Clara, USA), where the standard was set as RNA integrity number >7. RNA amplification, was performed using SMART Seq V4 Ultra Low Input RNA Kit (Takara Bio, Kusatsu, Japan), following the manufacturer’s protocol. TruSeq Stranded mRNA Library Prep (Illumina, San Diego, USA) was used for library construction. NovaSeq. 6000 (Illumina) was used for RNA sequencing. Processes from alignment to post-treatment of FASTQ data obtained by RNA sequencing was performed using STAR^29^ and Picard software (http://broadinstitute.github.io/picard/) according to TOPMed RNA-seq pipeline guidelines using the supercomputer system at Tohoku University’ medical-megabank institute. The consistency of RNA/DNA samples was confirmed by comparing RNA sequence data and genotype of genomic DNA. Samples with insufficient data or a low number of reads were excluded, which resulted in 15 active-phase CD patients and five active-phase ulcerative colitis patients for expression analysis. The number of reads of each transcript was calculated using the featureCounts (Ver 1.6.4)^30^ and were standardized against entire transcripts using edgeR (Ver 3.20.9)^31^. eQTL analysis and standardization at the gene level were performed using FastQTL (Ver 2.184) with–normal option^32^.

### GWAS

The GWAS data were analyzed with a linear mixed model. Genome-wide Complex Trait Analysis software (Ver 1.91.7b1) was used for the analysis^33^, where 7,424,691 polymorphisms with minor allele frequencies of over 0.5% were analyzed among the 16,919,636 polymorphisms input.

### TWAS

FUSION software was used for the TWAS^6^. The data for analysis consisted of RNA sequence data from whole blood, Epstein–Barr virus (EBV)-immortalized B cells, the transverse colon, the sigmoidal colon, and the small intestine (the ileum terminal), as these tissues are considered, among GTEx V7 data released in GTEx^5^, to be highly related to IBD.

### Statistical analysis

In eQTL analysis, samples showing *p* values less than 1e-06 were considered significant correlation. *p* values under 1e-04 were considered as candidates and were used for further analyses. In GWAS, polymorphisms showing *p* < 5e-08 in the linear mixed model were considered to be significant and those with p values under 1e-05 were considered to be candidates. Polymorphisms showing correlation within 500 kbps upstream and downstream of the polymorphism were considered cases of correlation at the same region. For TWAS, the genes with false discovery rate (FDR) of <0.05 were considered to be susceptibility genes, and genes with FDR < 0.10 were considered to be candidates. Data obtained from each analysis was further analyzed using R software (Ver 3.4.4). Supplementary Table S7 shows the eQTL data set analyzed in this study.

## Supplementary information

Supplementary information.

Supplementary information2.
