# Supplementary material for: An Integrated Genomic and Transcriptomic Analysis Reveals Candidates of Susceptibility Genes for Crohn’s Disease in Japanese Populations
Source: Sci Rep. 2020 Jun 24;10:10236. doi: 10.1038/s41598-020-66951-5 (PMC7314801; doi:10.1038/s41598-020-66951-5)
Supplement: Supplementary file 1 — Supplementary information. [file 41598_2020_66951_MOESM1_ESM.pdf]

Supplementary Materials

An Integrated Genomic and Transcriptomic Analysis Reveals Candidates of Susceptibility Genes for Crohn's Disease in Japanese Populations

Yoichi Kakuta[1], Ryo Ichikawa[1], Yuta Fuyuno[2], Atsushi Hirano[2], Junji Umeno[2], Takehiro Torisu[2], Kazuhiro Watanabe[3], Akihiro Asakura[4], Takeru Nakano[1], Yasuhiro Izumiyama[1], Daisuke Okamoto[1], Takeo Naito[1], Rintaro Moroi[1], Masatake Kuroha[1], Yoshitake Kanazawa[1], Tomoya Kimura[1], Hisashi Shiga[1], Takeshi Naito[3], Motohiro Esaki[2,5], Yosuke Kawai[6], Katsushi Tokunaga[6], Minoru Nakamura[7], Takayuki Matsumoto[2,8], Masao Nagasaki[4,9], Yoshitaka Kinouchi[10], Michiaki Unno[3], Atsushi Masamune[1]

[1] Division of Gastroenterology, Tohoku University Graduate School of Medicine, Sendai, Japan
[2] Department of Medicine and Clinical Science, Graduate School of Medical Sciences, Kyushu University, Fukuoka, Japan
[3] Department of Surgery, Tohoku University Graduate School of Medicine, Sendai, Japan
[4] Human Biosciences Unit for the Top Global Course Center for the Promotion of Interdisciplinary Education and Research (CPIER), Kyoto University, Kyoto, Japan
[5] Department of Endoscopic Diagnostics and Therapeutics, Saga University Hospital, Saga, Japan
[6] Genome Medical Science Project, National Center for Global Health and Medicine, Tokyo, Japan
[7] Clinical Research Center, National Hospital Organisation (NHO) Nagasaki Medical Center, Omura, Japan
[8] Division of Gastroenterology, Department of Internal Medicine, Faculty of Medicine, Iwate Medical University, Morioka, Japan
[9] Department of Integrative Genomics, Tohoku Medical Megabank Organization, Tohoku University, Sendai, Japan
[10] Student Health Care Center, Institute for Excellence in Higher Education, Tohoku University, Sendai, Japan

Supplementary figure legends

Figure S1. Manhattan plots of GWAS in Japanese CD patients

Red line indicates *p* = 5e-08, where genes above the red line were defined to have significant correlation. Blue line indicates *p* = 1e-05, where genes above the blue line were defined to have correlation of a candidate level. All genes showing significant correlation were located in two regions: one in the *HLA* region on chromosome 6 and the other upstream of *TNFSF15* on chromosome 9. Candidate levels of correlation were detected in a further 23 regions.

Figure S2. Integrated analysis of GWAS and eQTLs of effector memory T cells from LPMC

A total of 19 SNPs was overlapped between the 671 SNPs which showed more than a candidate level of correlation in GWAS and 22,632 SNP-gene pairs which showed more than a candidate level of correlation in eQTL analysis.

Figure S3. Correlation plots of SNPs in candidate regions that showed correlation by TWAS

Figure shows plots of polymorphism periphery of five genes (a. APOBEC3A, b. RAP1A, c. NPIPB9, d. WDR31, and e. IGLV3-29) that showed correlation by TWAS. Each dot indicates -log10 (p-values) before (gray) and after (blue) adjustment by gene (green) that showed correlation.

Supplementary Figure S1

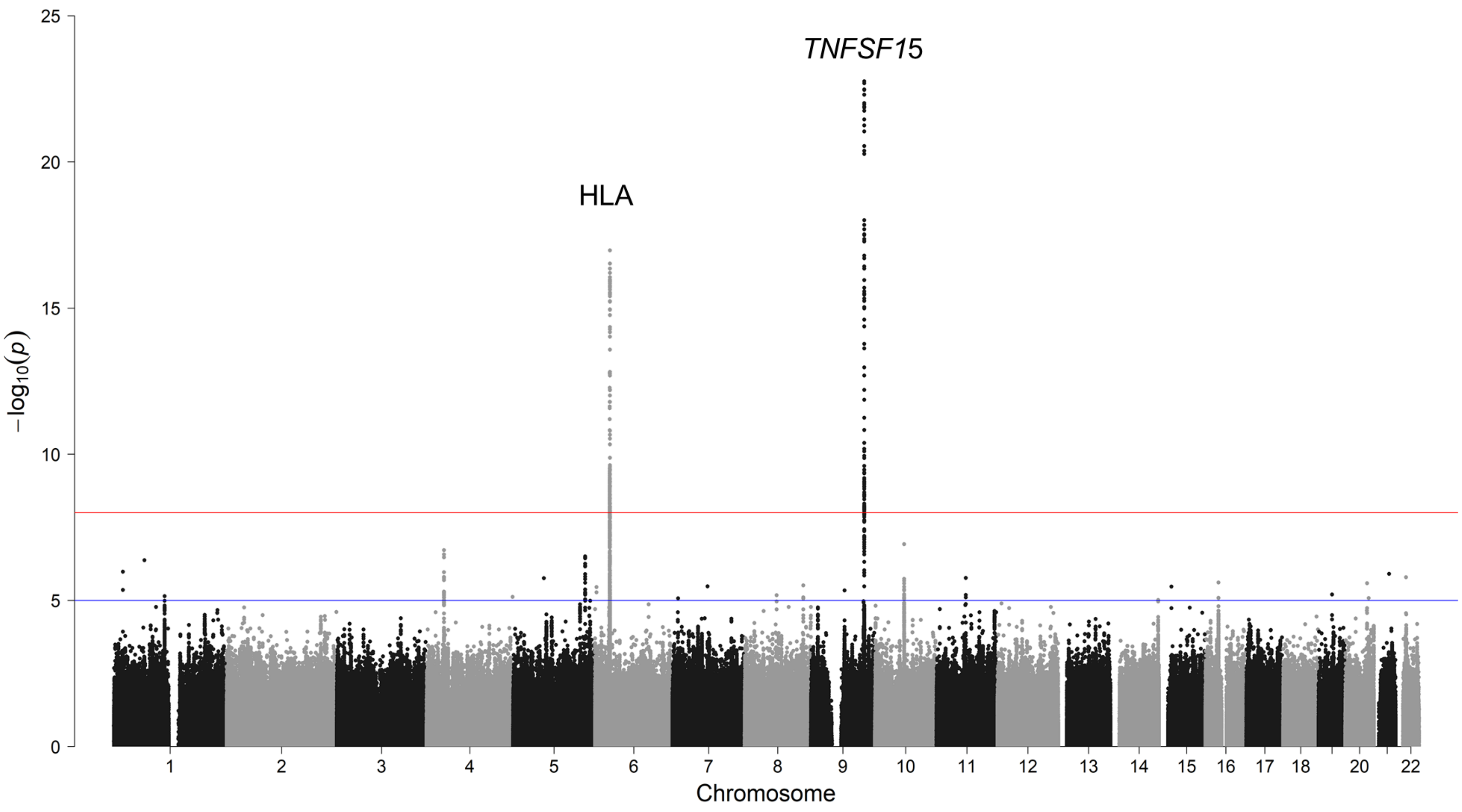

Supplementary Figure S2

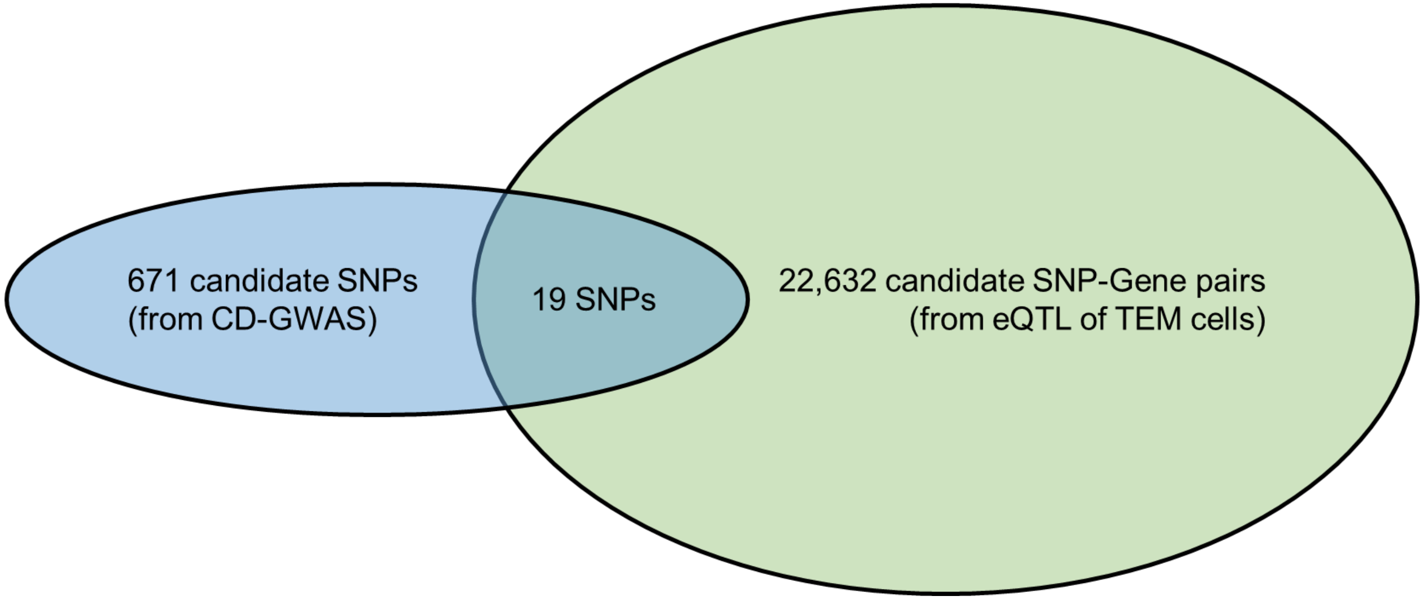

Supplementary Figure S3

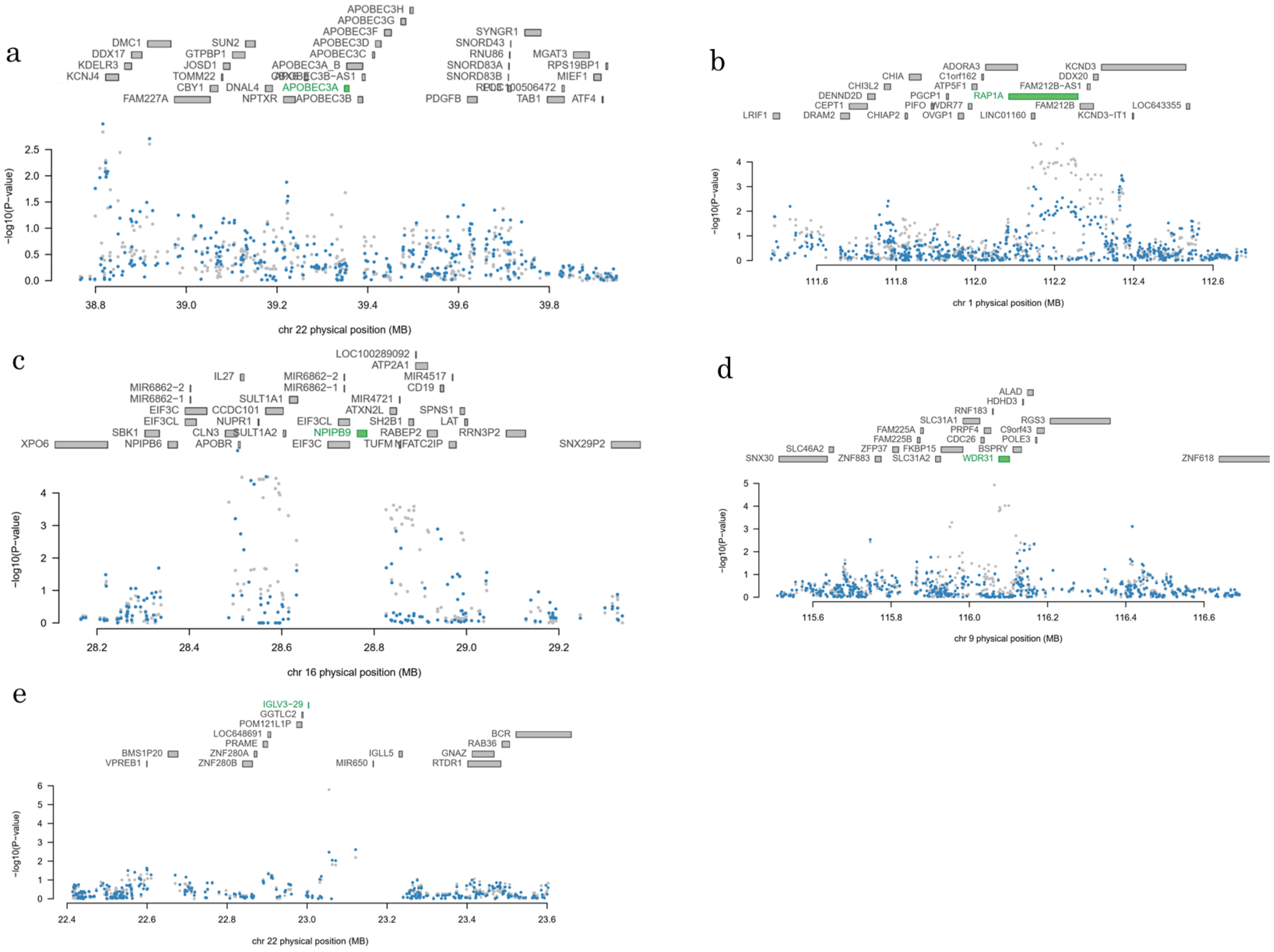

Supplementary tables

Supplementary Table S1. Significant eQTLs of effector memory T cells from LPMC

(Separate Excel file)

Supplementary Table S2. Candidate eQTLs of effector memory T cells from LPMC

(Separate Excel file)

Supplementary Table S3. Non-synonymous SNPs from CD-GWAS results

Chromosome	Position*	SNP ID	Gene	Exon	Function	A1	A2	A2 frequencies	p-values	OR(95%CI)
1	67648596	rs76418789	IL23R	exon4	p.Gly149Arg	G	A	0.053	4.22E-07	0.88 (0.84-0.93)
16	28513403	rs181206	IL27	exon4	p.Leu119Pro	A	G	0.083	3.28E-05	1.08 (1.04-1.12)
16	28603655	rs1059491	SULT1A2	exon7	p.Asn235Thr	T	G	0.074	4.38E-05	1.08 (1.04-1.12)

*Positions are based on the Genome Reference Consortium human build 37 (GRCh37)

Supplementary Table S4. Common SNPs between the CD-GWAS results and eQTL of TEM.

SNP							GWAS		eQTL		
Chromosome	SNP ID	Position*	A1	A2	A2 requencies	Distance to the gene	P_{GWAS}	OR(95%CI)	Gene	P_{eQTL}	Slope
6	rs117419513	32216166	G	A	0.121	239774	4.21E-06	1.09 (1.05-1.14)	TNXA	3.49E-05	1.17
6	rs117115819	32216355	T	C	0.121	239963	4.21E-06	1.09 (1.05-1.14)	TNXA	3.49E-05	1.17
6	rs28681257	32216934	A	G	0.121	240542	4.21E-06	1.09 (1.05-1.14)	TNXA	3.49E-05	1.17
6	rs4713514	32220741	A	C	0.121	244349	4.93E-06	1.09 (1.05-1.14)	TNXA	3.49E-05	1.17
6	rs117465038	32234981	T	A	0.109	258589	4.68E-06	1.10 (1.06-1.15)	TNXA	3.49E-05	1.17
6	rs117892681	32305814	C	A	0.103	329422	5.05E-06	1.10 (1.06-1.15)	TNXA	4.45E-05	1.20
6	rs117892681	32305814	C	A	0.103	329422	5.05E-06	1.10 (1.06-1.15)	TNXA	4.45E-05	1.20
6	rs117794128	32321833	A	G	0.121	345441	3.83E-06	1.10 (1.05-1.14)	TNXA	3.49E-05	1.17
6	rs117040717	32329731	T	C	0.120	353339	2.92E-06	1.10 (1.05-1.14)	TNXA	3.49E-05	1.17
6	rs3129915	32332264	G	A	0.127	355872	6.50E-07	1.10 (1.06-1.14)	TNXA	3.49E-05	1.17
6	rs3129916	32332322	G	A	0.127	355930	6.50E-07	1.10 (1.06-1.14)	TNXA	3.49E-05	1.17
6	rs3129924	32333299	C	T	0.127	356907	8.01E-07	1.10 (1.06-1.14)	TNXA	3.49E-05	1.17
6	rs3129925	32333419	G	A	0.127	357027	6.50E-07	1.10 (1.06-1.14)	TNXA	3.49E-05	1.17
6	rs3129926	32333480	T	C	0.126	357088	6.30E-07	1.10 (1.06-1.14)	TNXA	3.49E-05	1.17
6	rs2143462	32335204	G	A	0.127	358812	6.50E-07	1.10 (1.06-1.14)	TNXA	3.49E-05	1.17
6	rs2143461	32335347	G	A	0.127	358955	6.50E-07	1.10 (1.06-1.14)	TNXA	3.49E-05	1.17
6	rs3129935	32336205	A	G	0.127	359813	6.50E-07	1.10 (1.06-1.14)	TNXA	3.49E-05	1.17
6	rs3129937	32336364	C	A	0.127	359972	6.50E-07	1.10 (1.06-1.14)	TNXA	3.49E-05	1.17
6	rs117433623	32403963	C	G	0.108	427571	6.34E-09	1.13 (1.08-1.18)	TNXA	3.49E-05	1.17

*Positions are based on the Genome Reference Consortium human build 37 (GRCh37)

Supplementary Table S5. Summary of the TWAS with the susceptibility loci for CD in Japanese (HLA region)

Tissue	Gene	GWAS		eQTL			TWAS		
		Best SNP	Z-Score	SNP	R2	Z-Score	Z-Score	P-Value	FDR
Whole blood	HLA-DRB9	rs9271170	-6.08	rs9271170	0.16	-7.91	5.74	9.63E-09	2.86E-05
Whole blood	HLA-DRB6	rs9271170	-6.08	rs28366298	0.38	11.81	5.33	9.84E-08	1.95E-04
Whole blood	HLA-DQA2	rs9271170	-6.08	rs28366298	0.49	13.48	4.94	7.93E-07	1.18E-03
Whole blood	HLA-DRB1	rs9271170	-6.08	rs28366298	0.18	-8.27	-4.48	7.38E-06	8.76E-03
Whole blood	HLA-DQB1	rs9271170	-6.08	rs1063355	0.61	14.94	-4.13	3.64E-05	3.10E-02
Whole blood	HLA-DQB2	rs9271170	-6.08	rs1063355	0.58	-14.60	4.13	3.66E-05	3.10E-02
Blood - EBV-transformed lymphocytes	HLA-DRB6	rs9271170	-6.08	rs9271170	0.44	-7.62	5.16	2.52E-07	6.89E-04
Blood - EBV-transformed lymphocytes	HLA-DQA2	rs9271170	-6.08	rs1063355	0.62	-8.43	4.42	9.71E-06	9.02E-03
Blood - EBV-transformed lymphocytes	HLA-DQB2	rs9271170	-6.08	rs1063355	0.72	-9.11	4.42	9.90E-06	9.02E-03
Blood - EBV-transformed lymphocytes	HLA-DQB1	rs9271170	-6.08	rs1063355	0.67	8.81	-4.27	2.00E-05	1.37E-02
Blood - EBV-transformed lymphocytes	HLA-DRB5	rs9271170	-6.08	rs9271055	0.45	7.21	-4.11	3.92E-05	2.14E-02
Small Intestine - Terminal Ileum	HLA-DRB6	rs9271170	-6.08	rs9271055	0.38	-6.93	5.31	1.08E-07	3.07E-04
Small Intestine - Terminal Ileum	HLA-DQA2	rs9271170	-6.08	rs1063355	0.41	-7.59	5.06	4.20E-07	5.97E-04
Small Intestine - Terminal Ileum	HLA-DRB5	rs9271170	-6.08	rs9271055	0.45	7.42	-4.80	1.62E-06	1.54E-03
Small Intestine - Terminal Ileum	HLA-DQB2	rs9271170	-6.08	rs1063355	0.52	-8.04	4.08	4.49E-05	3.19E-02
Small Intestine - Terminal Ileum	HLA-DRB9	rs9271170	-6.08	rs9271055	0.25	-5.67	3.92	8.72E-05	4.96E-02
Colon - Transverse	HLA-DQA2	rs9271170	-6.08	rs28366298	0.42	10.72	5.46	4.67E-08	2.46E-04
Colon - Transverse	HLA-DRB6	rs9271170	-6.08	rs28366298	0.45	10.51	4.97	6.76E-07	1.78E-03
Colon - Sigmoid	HLA-K	rs9258158	-2.84	rs375984	0.12	-5.54	7.78	7.40E-15	3.57E-11
Colon - Sigmoid	HLA-DQA2	rs9271170	-6.08	rs28366298	0.51	10.19	4.91	9.34E-07	2.25E-03
Colon - Sigmoid	HLA-DRB6	rs9271170	-6.08	rs28366298	0.51	10.21	4.55	5.29E-06	8.50E-03
Colon - Sigmoid	HLA-DQB2	rs9271170	-6.08	rs1063355	0.55	-10.53	4.29	1.82E-05	1.91E-02
Colon - Sigmoid	HLA-DQB1	rs9271170	-6.08	rs1063355	0.58	10.87	-4.27	1.98E-05	1.91E-02
Colon - Sigmoid	HLA-DQA1	rs9271170	-6.08	rs1063355	0.25	7.21	-4.12	3.73E-05	3.00E-02

GWAS: genome-wide association study, TWAS: transcriptome-wide association study, FDR: false discovery rate

Supplementary Table S6. TWAS results of the candidate genes in other tissues

	TWAS P-value				
Gene	Whole blood	Lymphocytes	Small intestine	Transverse colon	Sigmoid colon
TNFSF15	2.28E-17	NA	NA	NA	NA
APOBEC3A	9.66E-05	NA	NA	NA	NA
ERV3-1	6.81E-04	4.79E-05	5.15E-03	1.54E-03	3.53E-03
RAP1A	2.66E-01	2.42E-04	NA	NA	NA
NPIPB9	NA	1.11E-02	NA	8.74E-05	4.78E-04
IGLV3-29	NA	NA	NA	1.29E-04	NA
ZNF713	3.92E-02	3.46E-02	NA	3.33E-02	4.41E-05
WDR31	NA	NA	NA	3.07E-04	1.34E-04

.

Supplementary Table S7. eQTL data used in this study

Tissue	Data Source	No. of samples	No. of genes
Whole blood	GTEx V7	369	6007
Blood - EBV-transformed lymphocytes	GTEx V7	117	2758
Small Intestine - Terminal Ileum	GTEx V7	122	2879
Colon - Sigmoid	GTEx V7	203	4874
Colon - Transverse	GTEx V7	246	5316
LPMC – Effector momory T Cell	this study	20	2463
